# Challenges in Diagnosing Necrotizing Sarcoid Granulomatosis: The First Case Reported From Indonesia

**DOI:** 10.1155/carm/3219868

**Published:** 2025-04-14

**Authors:** Gurmeet Singh, Ramadhan Karsono, Soedarman Sjamsoe, Muhammad Rizki Triono, Rina La Distia Nora, Devi Felicia, Fajar Lamhot Gultom, Daniel Ruslim, Arif Sejati, Ralph Girson Gunarsa, Ceva Wicaksono Pitoyo, Cleopas Martin Rumende

**Affiliations:** ^1^Division of Respirology and Critical Illness, Universitas Indonesia Departemen Ilmu Penyakit Dalam, Rumah Sakit Dr. Cipto Mangunkusumo, Jakarta, Indonesia; ^2^Mochtar Riady Comprehensive Cancer Centre Siloam Hospitals Semanggi, Jakarta, Indonesia; ^3^Department of Surgical Oncology, Rumah Sakit Dharmais Pusat Kanker Nasional, Jakarta, Indonesia; ^4^Jakarta Eye Center, Eye Hospitals & Clinics, Perhimpunan Dokter Spesialis Mata Indonesia (PERDAMI), Jakarta, Indonesia; ^5^Department of Ophthalmology, Rumah Sakit Dr. Cipto Mangunkusumo, Jakarta, Indonesia; ^6^Department of Anatomical Pathology, Mochtar Riady Comprehensive Cancer Centre Siloam Hospitals Semanggi, Jakarta, Indonesia; ^7^Department of Anatomical Pathology, Universitas Kristen Indonesia Fakultas Kedokteran, Jakarta, Indonesia; ^8^Department of Radiology, Universitas Tarumanagara Fakultas Kedokteran, Jakarta, Indonesia; ^9^Division of Cardiology, Department of Internal Medicine, Universitas Katolik Indonesia Atma Jaya Fakultas Kedokteran Dan Ilmu Kesehatan, Jakarta, Indonesia; ^10^Divison of Medical Hematology and Oncology, Department of Internal Medicine, Mochtar Riady Comprehensive Cancer Centre Siloam Hospitals Semanggi, Jakarta, Indonesia

**Keywords:** extrapulmonary sarcoid, malignancy, necrotizing sarcoid granulomatosis, thyroid, tuberculosis

## Abstract

Necrotizing sarcoid granulomatosis (NSG) is a rare disease characterized by granulomatous and necrotic features as well as vasculitis, and it primarily affects the lungs, with occasional extrapulmonary manifestations. The first documented case was in Jakarta, Indonesia. A 71-year-old male presented with prolonged fever, a neck mass, and multiple mediastinal lymphadenopathy. The disease was initially suspected as lung tuberculosis, but a surgical biopsy of the left thyroid lobe confirmed the NSG pattern. Treatment with oral prednisolone led to positive outcomes, as evidenced by radiological improvement at the 3-month follow-up. This case report aims to emphasize the challenges and the importance of clinician awareness in diagnosing NSG.

## 1. Introduction

Necrotizing sarcoid granulomatosis (NSG) of the lungs is a rare presentation of sarcoidosis. It was first reported by Liebow in 1973 and termed “sarcoidosis with NSG pattern” [1, 2]. This disease predominantly affects women across a wide age range, most commonly between 20 and 60 years [3]. To date, numerous case reports have described its primary manifestation in the lungs, though it can also involve other organs, including the liver, thyroid, and eyes [2–13]. The initial suspicion of NSG often arises from the presence of lung mass and nodules that were initially suspected as a case of malignancy [14]. NSG is typically diagnosed through histopathological examination of the affected organ tissue. Although the extrapulmonary manifestation of NSG has been previously reported, but the reports of NSG thyroid manifestation are rare. This case report presents the first case of extrapulmonary NSG in Jakarta, Indonesia.

## 2. Case Report

**Figure 1:**
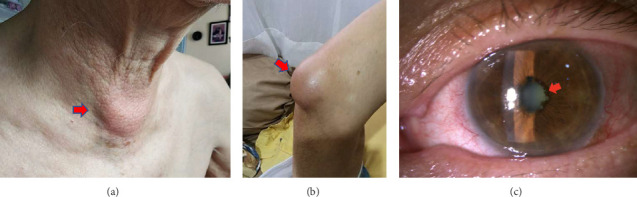
Clinical images of patients with enlarged masses in the neck region (photo was taken from the patient's next of kin two weeks before admission) (red arrow) (a). Mass in the right knee area (2022) (red arrow) (b). Granulomatous uveitis anterior of the left eye with posterior synechiae (2023) (red arrow) (c).

**Figure 2:**
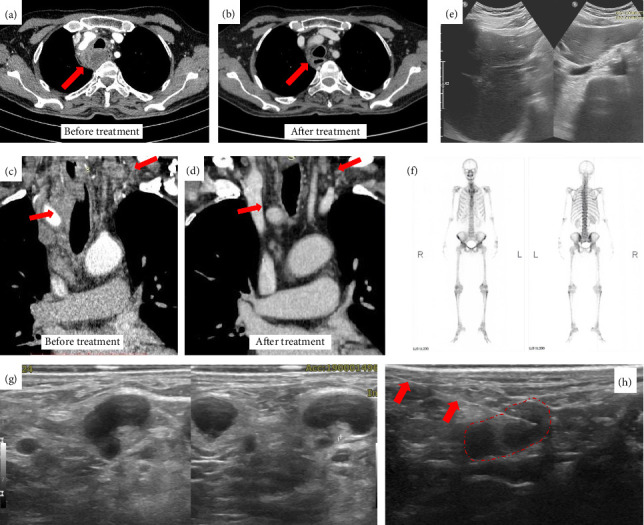
Imaging evaluation of the patients. Comparison of CT thorax from early 2024 (a, c) with CT evaluation after treatment (b, d) showed changes in the paratracheal mediastinal lymph nodes (a, b) and left base of the neck (c, d). The posttreatment scan showed a reduction in size and shift of the trachea and esophagus to the left side, compared to the previous enlargement and necrotic appearance. Posttreatment ultrasound evaluation showed improvement as the nodule was no longer visible in the liver (e). No pathological osteoblastic lesions are observed on the bone scan with Tc-99m MDP (f). Right posterior cervical lymph node FNAB (g). Ultrasound-guided right posterior cervical lymph node FNAB (h).

A 71 years old male was referred to our hospital in 2024 with intermittent fever, weakness, decreased appetite, and an enlarged mass in the neck region 2 weeks prior to admission (Figure 1(a)). In 2022, the patient reported similar symptoms, which subsided with antibiotic and anti-inflammatory treatment (Figure 1(b)). The patient had a history of cataract surgery in the right eye in 2022, secondary glaucoma, and acute granulomatous uveitis in the left eye in 2023 (Figure 1(c)). The inflammation subsided with high-dose oral methylprednisolone and 1% topical prednisolone acetate. The intraocular pressure was controlled with antiglaucoma medication and laser iridotomy. The patient's diabetes mellitus and hypertension were well-controlled. Lung tuberculosis was diagnosed in 2023, and chest computed tomography (CT) indicated fibrosis and bronchiectasis changes (mosaic attenuation), with multiple mediastinal lymphadenopathies (paratracheal and subcarinal). Antituberculosis drugs (ATDs) were introduced and then discontinued after 2 weeks due to hepatotoxicity. Although the patient's condition was stable for 2 months, intermittent fever and loss of appetite symptoms were resuscitated by the end of 2023. Subsequently, chest CT was conducted in early 2024 (Figures 2(a), 2(c)).

**Figure 3:**
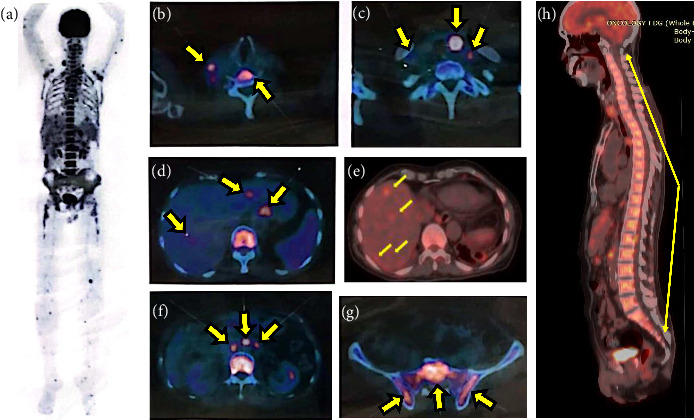
F-18 fluorodeoxyglucose positron emission tomography/computed tomography at the first admission to the hospital showed disseminated FDG uptake (a). A nodule capturing FDG was observed in the left lobe of the thyroid (SUV_max_ of 18.02) (b). Lymph nodes capturing FDG were present in the right superior jugular region (SUV_max_ of 2.37), multiple in the right supraclavicular region (SUV_max_ of 6.80), and both infraclavicular regions (SUV_max_ of 4.77 on the right, and SUV_max_ of 6.69 on the left) (b–c). Multiple lesions capturing FDG were scattered throughout the liver segments (SUV_max_ of 4.68) (d–f). A pathologically inhomogeneous FDG–avid pattern was observed in the spinal cord (SUV_max_ of 7.57) (g–h).

At presentation to our hospital, the patient was subfebrile and no enlarged mass was palpable in the neck region. Laboratory tests indicated Hb of 9.7 g/dL and WBC of 11,600 μL. The angiotensin-converting enzyme (ACE) test showed 7 U/L (reference: 9–67 U/L) and chest radiography revealed mild pulmonary fibrosis. Fluoroquinolones, antiemetics, and antipyretics were administered. Three days posttreatment, intravenous hydrocortisone (2 × 50 mg) was administered due to persistent fever. The clinical condition improved after 2 days of intravenous methylprednisolone. The results of 18F-fluorodeoxyglucose positron emission tomograph–CT (18F-FDG PET–CT) are presented in Figure 3. An advanced case of malignancy was suspected in this patient. A multidisciplinary discussion was conducted, and the decision was made to perform a left thyroid surgical biopsy, right posterior cervical lymph node fine-needle aspiration biopsy (FNAB), a liver biopsy, and an endobronchial ultrasound–guided transbronchial fine-needle aspiration (EBUSm–TBNA). The patient underwent a thyroid surgical biopsy and right posterior cervical lymph node FNAB. Echocardiography before surgical biopsy showed mild concentric left ventricular hypertrophy, Grade 2 diastolic dysfunction, and small pericardial effusion. The patient was discharged with oral prednisolone (2 × 5 mg).

**Figure 4:**
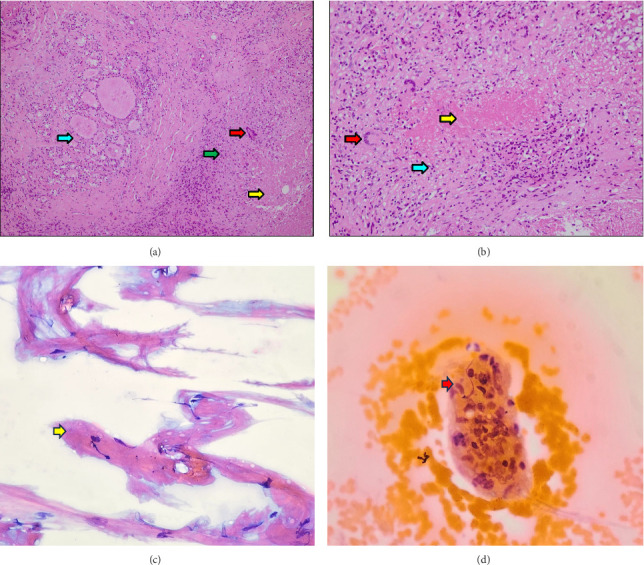
Histopathological findings of the thyroid tissue (a, b) and right deep cervical lymph nodes (FNAB) (c, d). Caseous necrosis (yellow arrow), multinucleated cells (langhans) (red arrow), lymphocytes, and histiocytes (green arrow) with unremarkable thyroid tissue in the background (blue arrow) were observed (a). Caseous necrosis (yellow arrow), multinucleated cells (langhans) (red arrow), and lymphocytes and histiocytes (blue arrow) are observed (b). The necrotic area (yellow arrow, (c)) and histiocytes (red arrow, (d)) are observed in the right deep cervical lymph nodes (FNAB).

**Figure 5:**
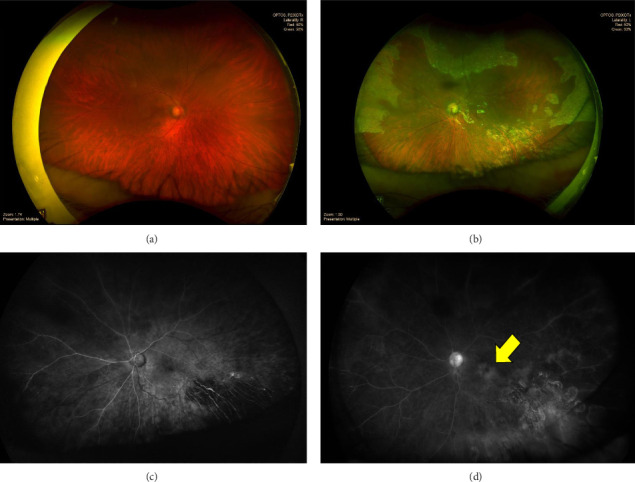
Ophthalmology evaluation showing ultra-widefield fundus photography of both eyes after vitrectomy of the left eye (a, b). Fundus fluorescein angiography showing minimal leakage in the macula (yellow arrow) (c, d).

Histopathological examination showed chronic necrotic granulomatous inflammation (Figures 4(a), 4(b), 4(c), and 4(d)), and acid-fast bacilli (AFB) with histochemical staining were negative. The patient showed good clinical response to prednisolone therapy and underwent another evaluation with fundus fluorescein angiography (FFA), echocardiography, and other imaging modalities. Echocardiography revealed a Grade 1 diastolic dysfunction with minimal pericardial effusion. Prednisolone therapy was continued for 3 months. Despite the positive response to prednisolone therapy, the patient experienced a recurrence of uveitis and eventually developed tractional retinal detachment. The traction was released by vitrectomy surgery, and the visual acuity improved (Figures 5(a), 5(b), 5(c), and 5(d)). Post three months of oral prednisolone therapy, the18F-FDG PET–CT evaluation showed a significant reduction in disseminated FDG uptake, with no visible FDG uptake in the left lobe of the thyroid, no multiple focal hypermetabolic lesions in the bones and muscles of both the upper and lower extremities, and decreased FDG uptake in both liver lobes. In addition, chest CT (Figures 2(b) and 2(d)), abdominal ultrasound (Figure 2(e)), and 3-phase bone scan with Tc-99m MDP (Figure 2(f)) revealed significant improvement. A second multidisciplinary discussion confirmed that the final diagnosis of this patient is NSG. The oral prednisolone dose was to be continued and adjusted to the clinical presentation of the patient.

## 3. Discussion

 Necrotizing sarcoid granulomatosis is a rare and controversial form of sarcoid-like granulomatous disease. Experts believed that the disease is in the same group as sarcoidosis, while others argued that it is sarcoidosis with an NSG pattern [2]. The incidence has been reported with an age range from 8 to 68 years, and the majority of patients were middle-aged (median = 42 years) [2, 4–7]. This is the first case reported in a 71-year-old male with clinical symptoms that presented atypically and mimiced a case of malignancy or infection. Nonproductive cough, fever, and dyspnea were the most common symptoms [15]. The most frequently observed radiological presentation was multiple nodules [12]. ACE levels were usually in the normal range and the CD4/CD8 ratio in bronchoalveolar fluid was often normal [13].

The disease was diagnosed based on the presence of a triad, namely, sarcoid-like granulomas, necrosis, and vasculitis, without any signs of an infectious cause. In clinical practice, clinicians specifically consider infectious etiologies in granulomatous pathology, such as tuberculosis, nontuberculous mycobacteria, or other infectious agents. Granulomatous inflammation morphology is often accompanied by inflammatory histological features (necrosis), and additional pathological examination, such as AFB histochemical staining using Ziehl–Neelsen staining, is needed. Previous reports have documented cases of this uncommon disease affecting areas outside of the lungs. Notably, the lymph node involvement beyond the chest cavity, especially in the thyroid gland, is an exceptionally rare occurrence [12]. In this case report, the patient was diagnosed with necrotic granulomatous inflammation features from left thyroid lobe surgical biopsy, recurrent vasculitis (uveitis) in the left eye, negative AFB histochemical staining, and a good response to oral prednisolone therapy. The challenges in diagnosing NSG in this patient were the atypical initial presentations, which led to a presumed diagnosis of tuberculosis and a suspected case of malignancy.

The prognosis of NSG is generally positive and responds appropriately to systemic steroid therapy [6], as shown in this case report that revealed improvement in both clinical and radiological features after treatment with oral prednisolone therapy.

In conclusion, extrapulmonary NSG is a rare entity that is challenging for clinicians to diagnose. It is crucial for clinicians to consider NSG as one of the differential diagnoses in patients with histopathological features of necrotizing granulomatous inflammation not responding to ATD treatment, particularly in endemic regions of tuberculosis.

## Data Availability

The data supporting the findings of this study are available from the corresponding author upon reasonable request.
